# Relationship Between Villous Atrophy and tTGA Levels in Dyspeptic Patients: A Case Series

**DOI:** 10.7759/cureus.15043

**Published:** 2021-05-15

**Authors:** Muhammad S Achakzai, Muhammad Samsoor Zarak, Zara Arshad, Hamaiyal Sana, Helmand Khan Tareen, Khushhal Khan, Aurangzeb Baloch, Saliha Kakar, Aqeel Nasim

**Affiliations:** 1 Gastroenterology, Bolan Medical College, Quetta, PAK; 2 Internal Medicine, Bolan Medical College, Quetta, PAK; 3 Medicine, Bolan Medical College, Quetta, PAK; 4 Pharmacy, University of Balochistan, Quetta, PAK

**Keywords:** villous atrophy, anti ttg, pakistan, gastroentero-hepatology, gastroenterology, clinica gastroenterology, endoscopy

## Abstract

Aim

The objective of the study was to observe the association of villous atrophy with anti-tissue transglutaminase levels in the identified subjects that met our addressed inclusion criteria.

Methods

A case series study was conducted among 40 patients presenting with dyspepsia along with signs and symptoms of celiac disease at the Bolan Medical Complex Hospital, Quetta over a period of five months from 25/5/17 to 25/10/17. The patients were referred to undergo tissue transglutaminase antibody serum test. The positive ones underwent biopsies to assess pathological entities including villous atrophy, blunting (focal or total), crypts, Intestinal layers and the number of Intraepithelial lymphocytes. The results collected were analyzed by using IBM SPSS version 20 (IBM Corp., Armonk, NY).

Results

There was a weak, negative correlation between tTGA and focal villous blunting (r = -0.345, p = 0.029) showing that high levels of tTGA are associated with lower risk of focal villous blunting. Correlation of tTGA and total villous blunting was a weak positive correlation (r = 0.282, p = 0.07) showing that high levels of tTGA are associated with increased risk of total villous blunting. There was a weak, negative correlation between tTGG and focal villous blunting (r = 0.409, p = 0.009) showing thathigh levels of tTGG are associated with a greater risk of focal villous blunting (p < 0.01) while tTGG and total villous blunting was a weak negative correlation (r = -0.330, p = 0.03) showing that high levels of tTGG are associated with lower risk of total villous blunting.

Conclusion

The study concludes by providing evidence of the absence of tissue transglutaminase antibodies in patients with histology-proven celiac disease. It implies that serology tests may be negative in some of the patients with typical chronic symptoms. Therefore, in such cases, histopathology may be conclusive in defining the status of celiac disease.

## Introduction

Villous atrophy is the condition of inflammation of intestinal mucosa [1], which results in loss of structural entity that in turn compromises the absorptive functions of the intestines. Villous atrophy has been proved to have high specificity for celiac disease [2]. Celiac disease is an autoimmune disease of the small intestine that is triggered by consumption of glutton present in wheat or some other related proteins found in rye, and barley [3]

On ingestion, this gluten shows some classic symptoms like chronic diarrhea, steatorrhea, and weight loss. Weight loss is due to malabsorption because of villous atrophy. Treatment with a gluten-free diet (GFD) would help to improve the symptoms.

The diagnosis for celiac disease is done on the basis of the presence of the human leukocyte antigen genetic factor, accompanied by positive biopsy and serological antibodies. Among these serological markers, IgA anti-tissue transglutaminase shows a sensitivity of more than 95% (CI, 77%-100%) and specificity also greater than 95% (CI, 1%-100%) [4]. It is considered to be the most reliable serological screening test (along with IgA anti-endomysial (EMA)) for celiac disease diagnosis [5]. But small intestinal biopsy still remains the gold standard for celiac disease diagnosis.

Villous atrophy is almost always attributed to the presence of celiac disease, which comes as no surprise because cases of villous atrophy other than celiac disease like giardiasis, irritable bowel syndrome, Helicobacter pylori infection, or cancer [2]. Celiac disease was first thought to be a condition that mostly affected white Europeans. But later on, research proved that the low prevalence of celiac disease in regions like Asia, Africa, South America, and the middle east was due to under-diagnosis [6]. So celiac disease is now considered to be distributed worldwide [7] and has been calculated to have a prevalence of 1% in the western world [8-10].

It has also been observed that women have a higher prevalence of celiac disease as compared to men showing a ratio of 1:2.8 [11]. The prevalence of celiac disease in Pakistan follows the same pattern as that of Europe, which is about 1% (1:100) [1]. But the exact prevalence rate of celiac disease in Pakistan is yet unknown [12]. It is an acknowledged fact that awareness of celiac disease among health professionals is poor, diagnosis delays are fairly common.

## Materials and methods

A case series was conducted among the patients that presented with symptoms to Bolan Medical University and Health Sciences Quetta from 25/5/17 to 25/10/17. We identified 40 cases of patients that were presented with dyspeptic symptoms, and also showed signs and symptoms of Celiac Disease. They were referred for a Tissue transglutaminase antibody serum test from the private laboratory of Agha Khan University and Hospital. The test was performed using the IBL transglutaminase IgA ELISA kit. Symptomatic patients simultaneously underwent a biopsy (gold standard for diagnosis) performed during endoscopy by Gastroenterologists of Bolan Medical University and Health Sciences. Finally, in the biopsy the pathologists examined the presence (or absence) of villous atrophy, and whether it was focal or total, crypts, the different layers of intestine and the number of Intraepithelial lymphocytes. The results thus collected were then inserted in IBM SPSS statistics version 20 (IBM Corp., Armonk, NY).

Each biopsy sample collected that showed villous atrophy was then categorized for focal or total VA. The patients that showed the signs and symptoms of giardiasis or irritable bowel syndrome were not included in the study.

The 40 cases that met our inclusion criteria of our study, were also asked about their family history, personal history and history of the relative symptoms they were experiencing along with villous atrophy.

## Results

Demographics

Table 1 shows demographic characteristics of patients. Maximum patients 14 (35%) enrolled were from age group 12-21. Both genders were equally enrolled in the study. Complaint of diarrhea was observed in 19(47.5%) patients. Compliant of anemia was reported in only 12 (30%) patients. Maximum patients 38 (95%) did not have the complaint of vomiting. It was also observed from the table that 13 (32.5%) patients were reported to have the complaint of dyspepsia. Out of 40 patient cases, only 1 (2.5%) patient was suffering from bloating, and only 3 (7.5%) were observed to have gone through weight loss. Complaint of abdominal pain was observed in 10 (25%) patients. Majority of patients 22 (55%) were reported to have epigastric pain.

**Table 1:** Demographical and clinical characteristics of the patients.

Demographic characteristics		Frequency	Percentage
Age groups	1-11	0	0
	12-21	14	35
	22-31	13	32
	32-41	8	20
	42-51	4	10
	52-61	1	2.5
Diarrhea	Present	19	47.5
	Not present	20	50
	Failed to mention	1	2.5
Anemia	Present	12	30.0
	Not present	27	67.5
	Failed to mention	1	2.5
Vomiting	Present	1	2.5
	Not present	38	95.0
	Failed to mention	1	2.5
Dyspepsia	Present	13	32.5
	Not present	26	65.0
	Failed to mention	1	2.5
Bloating	Present	1	2.5
	Not present	38	95.0
	Failed to mention	1	2.5
Weight loss	Present	3	7.5
	Not present	36	90.0
	Failed to mention	1	2.5
Abdominal pain	Present	10	25.0
	Not present	29	72.5
	Failed to mention	1	2.5
Epigastric pain	Present	22	55.0
	Not present	17	42.5
	Failed to mention	1	2.5

Characteristics of Endoscopic findings

Endoscopic findings show majority of patients 14(35%) have duodenal fissuring, the rest of the cases were observed to have duodenal fissuring accompanied by decreased duodenal folds height and/or decreased duodenal folds number. Majority of patients 38 (95%) showed an increased number of intraepithelial lymphocytes (Table 2).

**Table 2:** Endoscopic findings.

Endoscopic findings	Frequency	Percentage
Normal	11	27.5
Duodenal fissuring	14	35.0
Decreased height of duodenal folds	1	2.5
Duodenal fissuring x Decreased height of duodenal folds	3	7.5
Duodenal fissuring x Decreased number of duodenal folds	4	10.0
Decreased height of duodenal folds x Decreased number of duodenal folds	4	10.0
Duodenal Fissuring x Decreased height of duodenal folds x Decreased number of duodenal folds	2	5.0
Failed to mention	1	2.5

Histopathological findings

Maximum number of patients showed 27 (67%) focal villous blunting and 12 (30%) patients were observed to have total villous blunting (Table 3).

**Table 3:** Histopathological findings.

Histopathological findings		Frequency	Percentage
Increased intraepithelial lymphocytes	Present	38	95.0
	Not present	2	5.0
Focal villous blunting	Present	27	67.5
	Not present	1	2.5
	Inadvertently absent	12	30.0
Total villous blunting	Present	12	30.0
	Not present	28	70.0

Frequency of tTGA and tTGG

In view of the normal tissue transglutaminase levels below 18U/ml the tTg IgA and IgG levels above 18U/ml were calculated as positive for the presence of celiac disease in the patients. It was observed in the table that 25 (62.5%) patients were negative for celiac disease with respect to the tTGA levels. And 29 (72.5%) patients were negative for celiac disease with respect to tTGG levels (Table 4).

**Table 4:** Frequency of tTGA and tTGG. tTGA: tissue transglutaminase A; tTGG: tissue transglutaminase G.

Positive/negative status ratio of celiac disease with 18U/ml of tTGA and tTGG as cut off level		Frequency	Percentage
Positive/negative status ratio of celiac disease with 18U/ml of tTGA	Negative	25	62.5
	Positive	15	37.5
Positive/negative status ratio of celiac disease with 18U/ml of tTGG	Negative	29	72.5
	Positive	11	27.5

Association of tTGs (tTGA and tTGG) with focal villous blunting and total villous blunting

The relationship between tTGA and focal villous blunting and total villous blunting was investigated using Pearson product-moment correlation coefficient. Preliminary analyses were performed to ensure no violation of the assumptions of normality, linearity. Correlations were interpreted using the following criteria [13]. r = 0.10 to 0.29 or r = -0.10 to -0.29 small correlation, r = 0.30 to 0.49 or r = -0.30 to -0.49 medium correlation and r = 0.50 to 1.0 or r = -0.50 to -1.0 large correlation (Table 5).

**Table 5:** Correlation tTGs with villous blunting. *Correlation is significant at the 0.05 level; **correlation is significant at the 0.01 level.

Variable	Correlation coefficient	p-value
tTGA		
tTGA-focal villous blunting	-0.345	0.029*
tTGA-total villous blunting	0.282	0.078
tTGG		
tTGG-focal villous blunting	0.409	0.009**
tTGG-total villous blunting	-0.330	0.03*

Association of tTGA with Focal Villous Blunting and Total Villous Blunting

The relationship between tTGA and focal villous blunting was investigated using Pearson product-moment correlation coefficient shown in Table 5. There was a weak, negative correlation between tTGA and focal villous blunting (r = -0.345, p = 0.029) that shows with high levels of tTGA associated with lower risk of focal villous blunting.

Correlation of tTGA and total villous blunting is a weak positive correlation (r = 0.282, p = 0.07) that shows with high levels of tTGA associated with increased risk of total villous blunting.

Association of tTGG with Focal Villous Blunting and Total Villous Blunting

The relationship between tTGG and focal villous blunting was investigated using Pearson product-moment correlation coefficient shown in Table 5. There was a weak, negative correlation between tTGG and focal villous blunting (r = 0.409, p = 0.009) that shows with high levels of tTGG associated with greater risk of focal villous blunting which is statistically significant at p < 0.01.

Correlation showed tTGG and total villous blunting is a weak negative correlation (r = -0.330, p = 0.03) that shows with high levels of tTGG associated with lower risk of total villous blunting.

Association of tTGA and tTGG with histopathology

Table 6 defines the statistically significant result of the presence of positive histopathological findings in patients with negative lab results for tTGA. It is statistically significant in the case of focal villous blunting where it is found negative in more than 20 patients having negative results for the tTGs; however, focal villous blunting was seen on histopathology. The same is the situation with tTGG in focal villous blunting and total villous blunting that are present in patients with negative lab results for tTGG. 

**Table 6:** Association of tTGA and tTGG with histopathology. tTGA: tissue transglutaminase A; tTGG: tissue transglutaminase G.

		Positive for celiac disease	Negative for celiac disease	Total	p-value
Histopathology for tTGA					
Increased intraepithelial lymphocytes	Present	15	23	38	0.281
	Not present	0	2	2	
Focal villous blunting	Present	7	20	27	0.029
	Not present	8	5	13	
Total villous blunting	Present	7	5	12	0.075
	Not present	8	20	28	
Histopathology for tTGG					
Increased intraepithelial lymphocytes	Present	11	27	38	0.372
	Not present	0	2	2	
Focal villous blunting	Present	4	23	27	0.010
	Not present	7	6	13	
Total villous blunting	Present	6	6	12	0.003
	Not present	5	23	28	

## Discussion

This paper describes the relationship between villous atrophy and serum level of TGA in dyspeptic patients in the region of Quetta. Villous Atrophy is the condition of inflammation of the intestinal mucosa that causes gradual loss of its structural entity. And consequently, this damage to the mucosa causes compromised absorptive abilities and permeability of the intestine. Hence results in malnutrition (one of typical symptoms of celiac disease) [14].

According to the study previous done, it has been stated that villous atrophy has a very high specificity with celiac disease [2]. The study suggested the presence of villous atrophy in other diseases like giardiasis, irritable bowel syndrome, Helicobacter pylori infection or cancer. Out of our data almost all the cases with villous atrophy were diagnosed with celiac disease.

Celiac disease is an autoimmune disorder [15,16] that is also known as non-tropical sprue or gluten-sensitive enteropathy.

Celiac Disease is found in genetically susceptible individuals having either HLA-DQ2 or HLA-DQ8 genes coding mostly the small intestines. The immune response is generated as a result of release of Gliadin (alcohol-soluble portion of gluten) peptides that come from ingestion of wheat gluten, or its closely related species like barley and rye [17] and oat [18].

This gliadin reaches the intestinal epithelium's basal surface, and from there triggers the immune response using trans-cellular and/or para-cellular routes. The gliadin peptide has the amino acid sequence that has been proved to be specific for HLA-DQ2 (MHCII).

HLA-DQ2 and HLA-DQ8 are responsible for presenting this gliadin peptide to gluten-specific cells that can especially be found in the GIT of Celiac Disease patients. Tissue transglutaminase (tTG) responds to this gliadin by eventually forming glutamic acid and this process plays an important role in the T-cell recognition [19]. These T cells produced as a result of glutamic acid then start affecting the enzyme tTG in turn by producing anti-tTG antibodies from the mucosa of small intestine [20]. These anti-tTG antibodies can be found in the serological tests of patients suffering from Celiac Disease, and act as a very highly sensitive and specific marker for diagnosis of Celiac Disease. Although they disappear eventually if the patient is put on a GFD [21].

Both the presentation of Villous Atrophy and anti-tTG indicates towards the presence of celiac disease. According to the European Society of Pediatric Gastroenterology, Hepatology and Nutrition, the proper protocol of diagnosis of celiac disease regardless of its classical or non-classical symptoms [22] is first done by advising a serological test, in case of suggestive reports a biopsy is advised. The diagnosis can then be confirmed by putting the patient on GFD and assessing favorable response from serology and biopsy reports.

## Conclusions

The study provides evidence of the absence of tissue transglutaminase antibodies in patients with histology-proven celiac disease. The study that there is a possibility of having negative serology tests in symptomatic patients. The symptoms of chronic diarrhea, dyspepsia, and unintentional weight loss do not resolve and patients continue to visit healthcare providers. Therefore, in patients with typical symptoms, the celiac disease needs to be added in the differentials and proper work is needed. Even if the results of serological tests turn negative, histopathology may be conclusive in defining the status of celiac disease.
